# Learning and Its Neural Correlates in a Virtual Environment for Honeybees

**DOI:** 10.3389/fnbeh.2018.00279

**Published:** 2019-01-25

**Authors:** Hanna Zwaka, Ruth Bartels, Sophie Lehfeldt, Meida Jusyte, Sören Hantke, Simon Menzel, Jacob Gora, Rafael Alberdi, Randolf Menzel

**Affiliations:** ^1^Department of Biology and Neurobiology, Freie Universität Berlin, Berlin, Germany; ^2^Molecular and Cellular Biology, Harvard University, Cambridge, MA, United States; ^3^Bernstein Center for Computational Neuroscience, Berlin, Germany

**Keywords:** virtual enviroment (VE), learning and memory, mushroom body, honey bee, feedback neurons, GABA, operant learning, mushroom body extrinsic neurons

## Abstract

The search for neural correlates of operant and observational learning requires a combination of two (experimental) conditions that are very difficult to combine: stable recording from high order neurons and free movement of the animal in a rather natural environment. We developed a virtual environment (VE) that simulates a simplified 3D world for honeybees walking stationary on an air-supported spherical treadmill. We show that honeybees perceive the stimuli in the VE as meaningful by transferring learned information from free flight to the virtual world. In search for neural correlates of learning in the VE, mushroom body extrinsic neurons were recorded over days during learning. We found changes in the neural activity specific to the rewarded and unrewarded visual stimuli. Our results suggest an involvement of the mushroom body extrinsic neurons in operant learning in the honeybee (*Apis mellifera*).

## Introduction

In the past, two different approaches have been followed to search for neural correlates of operant learning and navigation: Monitoring neural activity of animals (usually rats) while navigating in a rather small space (O'keefe and Nadel, 1978; McNaughton et al., 2006; Puryear et al., 2010; Ball et al., 2014), and animals navigating in a virtual environment [VE, (Mallot et al., 1998)]. The latter has the advantage that the simulated environment can be large and fully manipulated. Its disadvantages relate to compromised sensory feedback provided by the moving visual world and the stationary conditions of the animal. Nevertheless, animals and humans can learn and navigate in a virtual reality set-up that produces the relevant visual feedback to the intended movements (Gillner and Mallot, 1998; Holscher et al., 2005, design guidelines for VEs can be found at: http://cogprints.org/3297/). Such devices have been developed and combined with neural recordings of EEG, local field potentials and single neurons for humans (Gillner and Mallot, 1998; Araújo et al., 2002; Baumeister et al., 2010; Doeller et al., 2010) as well as for animals (Mizunami et al., 1998; Harvey et al., 2009, 2012; Dombeck et al., 2010; Takalo et al., 2012; Aronov and Tank, 2014).

Insects are particularly suitable for behavioral-neural analyses. Despite having small brains they perform complex tasks in their respective environments (Menzel, 2012) including multiple forms of learning like classical, operant and observational learning under natural conditions. These forms of learning have been studied extensively on a behavioral level (Srinivasan, 2010; Giurfa and Menzel, 2013). Laboratory learning tests have been developed to keep the stimulus conditions close to those under natural conditions. In cockroaches, neurons were successfully recorded in freely moving animals (Mizunami et al., 1998; Bender et al., 2010). *Drosophila* flying in a simple VE has helped to elucidate a range of visual performances and visual learning at multiple levels of analysis (Heisenberg and Wolf, 1979; Wolf and Heisenberg, 1984; Peng et al., 2007). However, combining flight behavior in a VE with neural recordings has turned out to be rather difficult in *Drosophila* leading to correlations between turning behavior and local field potentials (van Swinderen and Greenspan, 2003) and to correlates of the fly's heading direction (Kim et al., 2017). Walking *Drosophila* are suitable for studying neural correlates of seemingly more complex behaviors like operant and observational learning (Fiala, 2007) and landmark orientation (Seelig and Jayaraman, 2015; Turner-Evans et al., 2017). These experiments allow researchers to combine the potential of molecular genetic tools with behavioral tests.

Honeybees are capable of a variety of complex learning tasks that go beyond classical and operant conditioning including non-elemental learning (Giurfa, 2003). So far, the search for neural correlates of learning, memory formation and memory retrieval in honeybees was limited to olfactory conditioning of restrained animals (Okada et al., 2007; Denker et al., 2010; Strube-Bloss et al., 2011; Menzel, 2014; Filla and Menzel, 2015). Past experiments have shown that honeybees can be trained to discriminate colors in a virtual environment (Buatois et al., 2017, 2018; Rusch et al., 2017; Schultheiss et al., 2017). Here we set out to train bees to visual stimuli in a virtual reality environment while recording simultaneously from higher order brain centers. In the future, this might enable us to record neural correlates of complex learning tasks in the virtual environment.

We developed a VE that consists of an air-supported spherical treadmill allowing the stationary walking honeybee (*Apis mellifera*) in closed-loop to control a visual environment projected onto a cone-shaped screen from above. This set-up gives us the opportunity to combine stable extracellular recordings over many hours with rather free moving animals. The honeybee can actively select a visual stimulus while we record from what are most likely A3 mushroom body extrinsic neurons that are known to change their response properties during classical olfactory conditioning (Haehnel and Menzel, 2010; Filla and Menzel, 2015). These neurons receive input from Kenyon cells, the intrinsic neurons of the mushroom body. They are sensitive to combinations of multiple sensory modalities including visual stimuli (Homberg and Erber, 1979; Schildberger, 1981; Grünewald, 1999).

Here, we also show that honeybees that were trained in free flight transfer the learned information to the VE.

After training in the VE, we found significant changes in neural activity to the rewarded and unrewarded colors in the VE.

## Experimental Procedures

### Spherical Treadmill, Geometry of the Virtual Environment and Overall Set-Up

The virtual environment (VE) was an advanced version of the VE described in deCamp (2013). The treadmill consisted of a Styrofoam sphere (10 cm diameter) placed in a half-spherical plastic cup with several symmetrically located holes through which a laminar airflow passed and let the sphere float on air.

The projector (Epson EMP-TW 700, Suwa, Japan, digital scanning frequency: pixel clock: 13.5–81 MHz, horizontal sweep: 15–60 kHz, vertical sweep: 50–85 Hz) was positioned above a Faraday cage and illuminated the inner surface of a cone-shaped screen (height 60 cm, bottom diameter 7 cm, top diameter 75 cm) via a large surface mirror and a Perspex window (Figure 2). The inner surface of the cone consisted of white paper. The shape of the patterns projected onto this screen were adjusted so that they appeared undistorted to the bee. During an experiment, the Faraday cage was closed. A web camera (c920, Logitech, Morges Gesellschaft, Switzerland) positioned above it imaged the head of the animal via a 500 mirror objective allowing observation of the animal during the experiment. The light from the projector, which fell directly on the upper view of the animal, was blocked by a screen.

### Control of the Virtual Environment and Experimental Procedure

The virtual environment and the recognition of the bee's movement was under the control of the custom program BeeWorld. It was implemented in Java by using OpenGL-Bindings for Java (LWJGL). Two optical high precision computer mice (Imperator, Razer Europe GmbH, Hamburg, Germany; G500, Logitech Europe S.A.) detected the movement of the sphere, initiated by the walking bee. The mice were accurately positioned under 90° at the equator of the Styrofoam sphere and precisely aligned to the optimal distance with x/y micro drives. The readings of the optical mice were precisely calibrated by rotating the sphere around the vertical axes. Thus, it was possible to convert ticks produced by the mice to forward movement, horizontal movement and rotatory movement of the bee in inch. Rotatory movement was obtained by calculating the mean of the movement along the x-axes of both mice. This redundancy is making the signal more reliable. The translational component of the movement results from the differences between the ticks of the y-axes of both mice. Both y-signals were multiplied with their position vectors and summed up. The resulting vector gives the translational components of the movement (Figure 2C):

$$Translation=x_{1}*\left(\begin{array}{c} -0.707\\ -0.707 \end{array}\right)+x_{2}*\left(\begin{array}{c} 0.707\\ -0.707 \end{array}\right)$$

To obtain the new position of the bee after a move, the translational vector was added in the direction the bee was virtually orientated:

$$\begin{array}{l} \begin{array}{c} T\text{.}x*\text{cos}\left(O\right)+T\text{.}y*\text{sin}\left(O\right)=x\\ T\text{.}y*\text{cos}\left(O\right)+T\text{.}x*\text{sin}\left(O\right)=y \end{array} \end{array}$$

T is the translation vector. O is the orientation.

The data from the mice was read at a frequency of 500 Hz by the computer. The bee was able to control the virtual scenery by rotatory and translatory movements of the sphere. Multiple scenarios were designed and stored as xml files. These files were loaded by the custom program BeeWorld. They contained the positions, widths, and colors (RGB) of a variable number of vertically oriented stripes or other structures. In order to improve the feedback to the bee about the rotatory components, every scenario had a checkerboard pattern projected onto the ground immediately in front of the bee, and gray stripes of different height at the background simulating a far-distant skyline. The rotation and translation of the checkerboard pattern was coupled one-to-one to the intended movement of the animal and the rotation of the skyline was set to a lower angular velocity simulating further distance by motion parallax. The angular velocities of these patterns as well as that of all objects in between could be separately adjusted.

The field of view in OpenGL was limited to 179°, the scenarios projected onto the screen, however, simulated a 360° view. To solve this, four 90° views were combined and transformed to fit the cone-shaped screen. In addition to the 360° view, the checkerboard pattern was projected in the middle of the screen. Movement and rotation speed of our 360° skyline and the checkerboard ground pattern was set relative to the rotation speed of the stripes in the scenarios. The depth components between virtual objects seemingly in the background and virtual objects closer to the animal were simulated by occlusion, size of the objects, and motion parallax that changed with movement of the animal.

Data from walking traces were synchronized with the data from spike recordings as collected with an analog/digital converter (micro3, CED, Cambridge Electronic Design, Cambridge, UK, 20 KHz sampling frequency per channel). A photodiode directed at the projector detected a short light signal under the control of the BeeWorld program and fed it into the ADC input of the analog/digital converter for synchronization. The scenario used in all experiments here consisted of one blue and one yellow vertically oriented stripe with an angular width of 30°, the horizontal checkerboard pattern immediately in front of the bee and the gray skyline pattern in the background. The angular rotation of the checkerboard pattern was equal to the angular movement of the sphere simulating a respective movement of the floor directly below and in front of the bee. The angular rotation of the stripe pattern was set to 75% of the checkerboard pattern, and a skyline pattern projected onto the screen together with the stripe pattern moved with 50% of the checkerboard pattern. Thus, these three patterns simulated different distances to the honeybee.

### T-Maze Experimental Design

Free-flying bees were trained in a T-maze (70 cm long until T-junction, about 5 cm in width, 4 cm high with a 55 cm long head side, see Figure 3) with one color (either blue or yellow) rewarded with 30% sucrose and another color punished with a 0.5 M potassium chloride solution. To attract foragers, a feeding station offering 1–10% sucrose solution was placed near the experimental set-up. During the first foraging flights, animals were actively induced into the entrance of the T-maze with a help of a sucrose-containing syringe. Individual foragers were marked, and all T-maze approaches were noted. Only one bee was tested at a time. Bees could fly into the T-maze but had to walk until a point of decision and decide for one side. In the beginning, sucrose droplets in the entrance of the maze showed the direction to the point of decision. The experimental set-up consisted of a plastic T-maze covered with UV-transparent Plexiglas to ensure daylight conditions within the maze. Little doors inside the maze ensured that the bee had to walk in one direction after the point of decision and could not turn back in order to perceive the colors again. The animals were trained over 25 trials.

Afterwards, they flew back to their hives or—in case of a wrong decision—could enter again. To avoid side preferences, we switched the sides of reward and punishment. Subsequently the animals were transferred to the virtual environment and tested in a scenario similar to the T-maze situation without reward.

### Animals

Worker honeybees (*Apis mellifera carnica*) were caught at the hive entrance during summertime. In winter, sugar water foraging animals flying in an indoor flight room were collected at the feeding sites. The bees were immobilized by cooling, mounted in Plexiglas tubes, and kept in a high humidity chamber. During the night, bees were held on Styrofoam spheres and were able to move freely on the spheres. All animals were fed to saturation after capture and on each subsequent day at 4 p.m. with 16 μl of 30% sucrose solution.

### Treadmill Training

During the night, bees were on Styrofoam spheres held in an apparatus to measure walking activity that only allowed the animal to walk forward and backward. The animals were able to move on the spheres held by balances, which kept the animal on the spherical treadmill with its own weight. This balance allowed the animal to also change the distance to the surface of the treadmill during walking. Walking activity was detected by means of walking distance. The walking distance was measured with light barriers that assessed the light shining through a wheel connected to the moving sphere with differently colored (black, gray, or transparent) subdivision. The percentage of measured light gave information about the turning direction and speed when measured over time. A connected Arduino microprocessor sent counted subdivision crosses per time to a computer that analyzed the data. Animals that performed well were later transferred to the VE.

### Virtual Environment Experimental Design

To study conditioning in the VE we allowed the animal to walk freely through the VE. Two different training paradigms were used in the VE. Either a color was rewarded (color-only) or a color was rewarded in combination with an odor (color-odor).

The animal was rewarded with 30% sucrose solution in the acquisition phase in both paradigms after arriving at a previously determined color. The colored stripe had to have a particular size and location in front of the animal to be rewarded (rewarding site). When the animal walked toward one stripe, the stripe turned in front of it depending on the rotatory movement of the spherical treadmill. When it kept walking toward the stripe (translatory movement of the sphere), it got bigger, simulating the object getting closer, and when it reached it, it covered 180° of the screen. Arriving at one color (10 cm walking distance) was counted as a decision. A custom-built rewarding device automatically turned toward the animal. This device consisted of a metal arm with an angle bracket at the end, holding a droplet of sucrose solution. Via Spike2 a digital command was sent to an analog-digital converter that sent a five-volt signal to a rotating motor (XFLY 400, Motraxx, Burgthann, Germany) that turned the arm toward the animal, so that it could reach the droplet. The honeybee could lick the sucrose solution for 10 s, either in combination with an odor or without. Afterwards the arm turned back automatically. Only in the color-odor paradigm, was the odor presented during the rewarded color for 4 s followed by a sucrose reward for 10 s with an overlap of 1 s. The odor was switched on when the animal reached the rewarding site. Spike2 controlled the odor stimulation, marked the time and sent a digital command, which triggered a relay board that in turn controlled the valves for odor stimulation. The odor pulse lasted for 4 s. In both designs, the color was switched off simultaneously with the ending of the reward. The reward lasted for 10 s. When the animal walked toward the unrewarded color (C–) nothing happened, and it could continue walking in the VE until it reached the rewarded color (C+). If the animals stopped walking for more than 20 min during the experiment, we switched to a different acquisition trial presenting the CS+ color, the odor and the US. All animals received at least eight acquisition trials including one to eight trials actively walking. The inter-trial interval was 10 min. For every animal, at least five trials were successful rewarded trials. Thus, we used the minimum of five trials for comparison between animals.

In a pre-test before the acquisition started, the naïve responses toward the stimuli were tested three times. Five stimuli were presented independently (see also Figure 5) in a pseudo-random fashion with an inter-trial interval of 1 min independent of the animal's movement.

The following terminology was used for the various training and test conditions:

- **CC**: We presented a stimulus for 14 s consisting of a blue and a yellow colored stripe. The stimulus was equal to the scenario that was presented at the starting point in every acquisition trial. This was the moment when the bee could walk toward one or the other color. Therefore, we will call this stimulus CC for color choice.
- **C+**: In this situation we showed the rewarded color (C+) alone for 14 s to the animal. In the test situation no reward was presented.
- **C–**: Here we presented the unrewarded color (C–) alone to the honeybee for 14 s. If the animal approached this color it experienced “no reward.” This happened both during acquisition and during the test.
- **US**: Additionally, we presented the sucrose reward alone (US) three times for 10 s to the animal.
- **O**: We presented the odor alone (O) three times in the pre-test for 4 s.

During training, the odor was only present when the animal approached the rewarded color. The odor was paired with the sucrose reward for 1 s.

In a memory test on the day after the acquisition (post-test), all color stimuli (CC, C+, and C–), the reward alone (US) and the odor alone (O) were presented to the honeybee again. This remained the only test situation in the experiment.

### Learning Performance in the VE During Extracellular Recording

The behavioral response evaluated as a measure of learning was the approach to the rewarded color or color/odor combination. The time until the animalreached the rewarded color was the measure of the learning effect (**Figure 6A**). As not all animals received five operant acquisition trials, we excluded one animal from the analysis.

### Electrophysiology

#### Extracellular Recording

For extracellular recordings, custom-made tetrodes consisting of four polyurethane-insulated copper wires were used (15 μm diameter, Elektrisola, Germany). The wires were twisted and glued together with superglue or by short exposure to 210°C. The tips were cut and electroplated, reducing the impedance to 80–150 kΩ (Ferguson et al., 2009) (Redish Lab, University of Minnesota) using the electroplating device NanoZ (Neuralynx, Bozeman, Montana).

#### Preparation of the Animals

A small piece of copier transparency film was fixed with dental wax on the thorax as a holder for the stationary running animal on the treadmill. Dissection started by opening the head capsule. Bee Ringer [NaCl (130 mM), KCl (6 mM), MgCl_2_ (4 mM), CaCl_2_ (5 mM), glucose (25 mM), sucrose (170 mM, pH 6.7)] was applied to the brain when necessary to avoid drying of the brain surface. The tip of the electrode was dipped in a 5% tetramethylrodamine-biotin solution (TMR, Microruby, MoBiTec, Göttingen, Germany) in 0.2 and 1 M potassium acetate. After recording, the recording site was verified in the dissected brain. Only animals with recordings from the desired recording site were admitted to the analysis.

The tip of the electrode was mounted on an external micromanipulator as described in Duer et al. (2015). The electrodes were inserted at the rim of the vertical lobe at a depth of 40–100 μm, where A3 neurons bifurcate and enter the vertical lobe. A silver-wire reference was coiled with the electrode, bent as a hook, and placed gently on the brain surface. After placing the electrodes in the selected brain area (ventral aspect of the mushroom body) under visual control, the electrode was fixed with non-toxic two-component silicon (kwik-sil, WPI, Sarasota) in the brain. After hardening of the kwik-sil, the electrode was released from the micromanipulator and the bee was transferred to the virtual environment set-up and carefully adjusted on the floating sphere. The electrode holder consisted of a small balance that kept the animal on the treadmill with its own weight. This balance also allowed the animal to change the distance to the surface of the treadmill during walking. The direct light from the LCD projector was shaded to prevent direct illumination of the dorsal regions of the compound eye and the ocelli. A manipulator made it possible to precisely center the animal on the spherical treadmill.

#### Analysis

Only animals that completed the whole experiment including pre-test and acquisition on the first day and a memory post-test on the second day were analyzed.

Single unit activity in extracellular recordings is not equivalent to neuronal activity as for example from single cells in intracellular recordings. In such an analysis, false-positive spikes might be detected, as there is always a potential risk of misclassification. False-positive errors are events that are wrongly classified and belong for example to a different cell. Contrarily false-negative errors are events that are misclassified as noise. Thus not all spikes from one cell are in one group (Harris et al., 2000; Joshua et al., 2007). However, automatic or semi- automatic spike sorting algorithms as used in this study reduce these errors close to an optimum between 0 and 8% (Harris et al., 2000).

**Figure 1:**
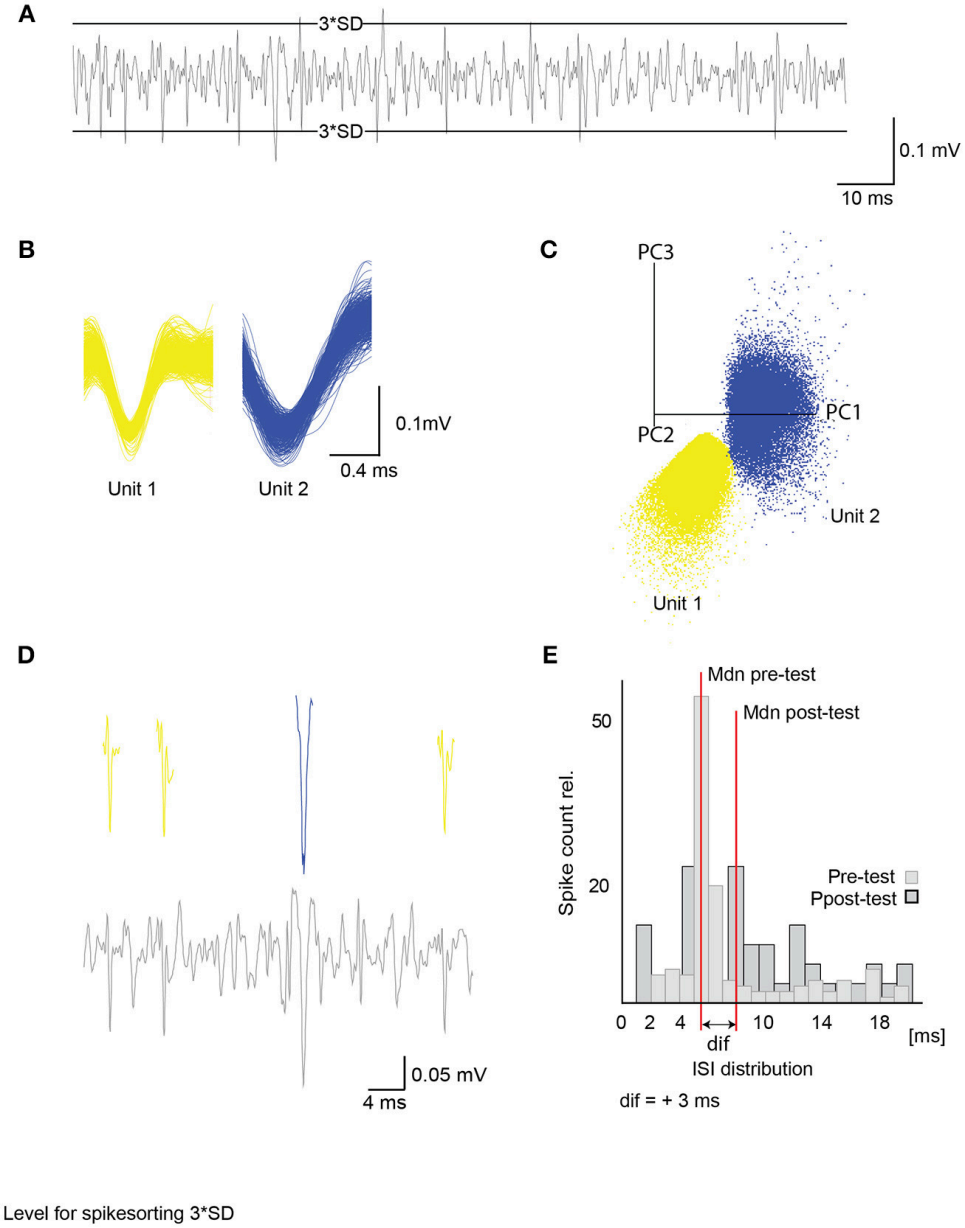
Unit activity extracted from raw data and inter-spike interval analysis. **(A)** Differential extracellular recording with a 3 times standard deviation (3^*^SD) level for spike sorting. Only activity crossing the levels was considered in spike detection. **(B)** Example of two single unit waveforms (500 events) with their corresponding principal component analysis. Axes give first 3 principal components **(C)**. **(D)** Example of a filtered extracellular recording channel (lower panel) with detected units from **(B)** (upper panel). **(E)** Example of a single unit inter-spike interval distribution for 600 ms after stimulus onset with relative spike count and inter-spike interval in milliseconds (ms). Median (Mdn) depicted in red is used to compare ISI before and after acquisition of single units with a Wilcoxon signed-rank test (see Figure 9). ISI, inter-spike interval; SD, standard deviation.

The data acquisition-and-analysis software Spike2 was used for the semi-automated template-matching spike sorting. All channels were recorded at a sampling rate of 20,000 Hz and low-pass filtered. One channel was differentially recorded from two electrodes, and two channels were single-ended electrodes. Before analysis, the data was filtered with a band-pass FIR-filter (350–2,500 Hz). The channel with the best signal-to-noise ratio was used for further analysis. Subsequently, the mean activity and standard deviation (SD) were calculated and used as a threshold for computing templates. Only events that either exceeded three SD or dropped below three SD were used for template matching (Figure 1A). In addition to this restrain, after sorting, all results were visually controlled for sorting quality and additionally checked by the first three components of each unit using a principal component analysis (PCA) provided by Spike2 (Figures 1B,C, Figure S1). Sorting results were improved using the K-means clustering approach provided by Spike2. Units that were present at the beginning of the experiment but decreased drastically until vanishing in the following 16 h were excluded from further analysis.

To identify rate changes to a stimulus in single unit activity we constructed peri-stimulus time histograms from all trials using a bin size of 50 ms. Afterwards we calculated the mean and the standard deviation (SD) from 20 s before stimulus onset. Rate changes that either exceeded the mean plus three times SD or dropped below the mean minus three times SD were considered an effect (Strube-Bloss et al., 2011).

In addition to changes in unit activity to the stimulus, we compared activity of single units in pre-tests, training, and 16 h tests. Thus, we analyzed changes in spike firing rates (SFR) from pre-test to acquisition and from pre-test to test toward the tested stimuli (Figures 9A,B). We calculated the ΔSFR using the activity extracted from the analysis for single unit changes toward the stimulus (compare Figure 5) exceeding the 3^*^SD threshold in the PSTHs. The spike activity was normalized by taking the ratio between stimuli response in pre-test and training or pre-test and test. Thus, we defined ΔSFR as the change in spike firing rate from e.g., pre-test to test toward a stimulus divided by total response:

$$\begin{array}{l} \Delta SFR=\frac{SFRtest-SFRpre}{SFRtest+SFRpre} \end{array}$$

A positive ΔSFR means an increase in neuronal firing, a negative ΔSFR means decreased neuronal firing, and ΔSFR of 0 means no change in neuronal firing due to the stimulus (Hussaini and Menzel, 2013). ΔSFR was calculated for every single unit. ΔSFRs of all units were analyzed together. We performed a Wilcoxon signed-rank test against a hypothetical change of zero.

To detect subtle excitatory or inhibitory rate changes that extend over several 100 ms to a stimulus, we compared the inter-spike interval (ISI) in the pre-test in a response window of 600 ms after stimulus onset- to the ISI in the post-test in a response window of 600 ms after stimulus (Figure 1D). The same was done during acquisition from stimulus to stimulus to search for ISI changes. We tested for significant differences in the median of the ISI distributions in all trials using a Wilcoxon singed-rank test. These approaches were adapted from an analysis of MB extrinsic neurons in Strube-Bloss et al. (2011). For depiction of the change, we subtracted the median ISI in the post-test from the median ISI in the pre-test for every single unit.

### Histochemistry

Brains were fixed with 4% paraformaldehyde (PFA, Roth, Karlsruhe, Germany) or a mixture of 1.3% PFA and 0.7% glutaraldehyde (GA, Sigma-Aldrich, Munich, Germany) for at least 4 h. Subsequently, brains were washed three times in phosphate buffered saline [PBS; NaCl (37 mM), KCl (2.7 mM), Na_2_HPO_4_ (8 mM), KH_2_PO_4_(1.4 mM), pH 7.2] for 10 min each, dehydrated in an ascending alcohol series (50, 70, 90, 99, and 100% each 10 min), cleared in methylsalicylate (Roth, Karlsruhe, Germany) for 10 min, and mounted on a special object slide (a metal plate of 0.5 mm thickness with a central hole and cover slips on both sides) in methylsalicylate.

### Confocal Imaging

Confocal image stacks of the whole brains were acquired using a confocal laser scanning microscope (Leica TCS SP2, Wetzlar, Germany) using a 40 × 0.4 IMM lens objective or a 20 × 0.5 water lens objective. Per stack, around 400 sections were scanned with a resolution of 1024 × 1024 voxels each, and with a voxel size of 0.61 × 0.61 × 1.3 μm or 0.73 × 0.73 × 1.1 μm. Stained neurons were scanned at 633 nm. Linear intensity compensation was used to adjust differences in brightness depending on scanning-depth.

### Statistics

For statistical analysis of multiple comparison, a Wilcoxon signed-rank test was performed (Statistica version 8.0, StatSoft, Inc., Tulsa, OK, USA). For normally distributed data, a two-way ANOVA was used to analyze the interaction between two variables (Statistica version 8.0, StatSoft, Inc., Tulsa, OK, USA) with an additional Fisher LSD *post-hoc* test. For comparison of nominal paired data, a McNemar test was performed in (R Core Team, 2017) as well as a binomial test. A two one-sided *t*-test (TOST) for equivalence was performed in (R Core Team, 2017) using the equivalence package for a paired sample with epsilon for small sample size = difference in standard deviations. In all tests, differences were considered significant if *p* ≤ 0.05.

### Terminology

The terms used to describe structural components of the honeybee brain have been adapted according to the nomenclature system of the Insect Brain Name Working Group (Ito et al., 2014).

## Results

### The Virtual Environment

**Figure 2:**
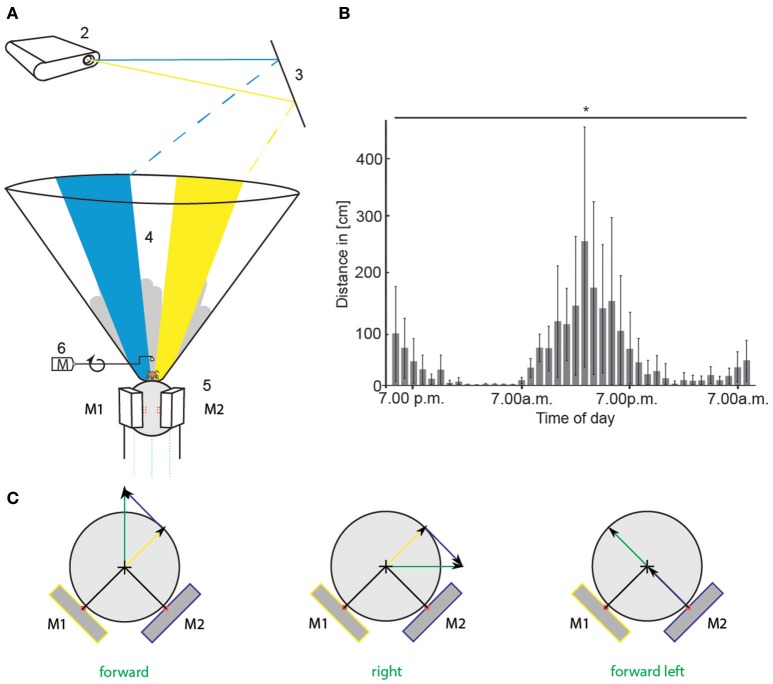
Virtual environment experimental set-up and circadian walking activity. **(A)** A honeybee was placed in the center of the VE set-up and ran on a Styrofoam sphere floating on air (1). An LCD projector (2) projected a scenario consisting of two colored stripes (blue and yellow), and a skyline via a mirror (3) onto the inner surface of cone-shaped screen (4). Two optical mice (5, M1 and M2) recorded the movement of the sphere when the bee walked. The scenario in the VE moved accordingly (closed-loop). The gray skyline in the background moved with different parallaxes than the colored stripes to mimic far-distant objects. A checkerboard projected directly on the sphere in front of the animal moved with the bee's walking speed providing direct visual-motor feedback. The scenario at the starting point of every operant learning trial is shown here. Two colored stripes were present. We call this stimulus color choice (CC). When the animal walked toward one stripe, the stripe moved in front of it. When it kept walking toward the stripe, it got bigger, simulating the object getting closer. Upon reaching it, it covered 180° of the screen. A rewarding device (6) turned toward the animal. It consisted of a metal arm on a motor (M) with an angle bracket at the end, holding a droplet of sucrose solution. **(B)** Circadian walking activity of 16 bees that were hold on similar spheres as in the VE for 40 h. Walking distance in cm per 1 h is plotted against daytime. Walking distances differed significantly between the hours [ANOVA, *F*_(39, 600)_ = 2.3, *p* < 0.0001]. Bars indicate standard deviation (SD). **(C)** Sketch of translatory input to computer mice (M1, M2) for different walking directions of the bee (green arrow). Depicted are the two mice in yellow and blue and their respective input in yellow and blue. Input of each mouse is used to calculate vectors (yellow and blue arrow) for reconstruction of the bee's walking trajectory (green arrow). VE, virtual environment. Asterisks indiacte statistical significance.

The virtual environment (VE) simulated a simplified 3D world for a honeybee walking stationary at the center of an air-supported spherical treadmill (Figure 2A). An LCD projector projected the visual patterns from above via a mirror into the inner surface of a cone. The distortion of the patterns caused by the projection on the inner surface of the cone were compensated for by the design of projected patterns. The depth components were simulated by occlusion, size, and motion parallax. The rotatory and translatory movements of the treadmill were translated into respective changes of the visual patterns. Different scenarios were written in a custom program implemented in Java by using OpenGL-Bindings for Java (LWJGL). We used a simple set of stimuli consisting of two colored stripes (blue and yellow) appearing in front of a structured gray skyline in the background, which moved with different parallaxes mimicking far-distant objects. To simulate the movement with the bee's actual walking speed, the animal walked on a checkerboard pattern projected directly onto the sphere in front of it, thus providing a direct visual-motor feedback by the ground over which the animals walked. Two optical mice recorded the movement of the sphere. To adapt to the walking situation, the experimental animals were held on similar spherical treadmills during the night. Animals on the spherical treadmills showed circadian activity (Figure 2B). All data reported here came from experiments at times of the day at which free-flying honeybees usually performed foraging behavior and accordingly the highest activity on the treadmill.

### Animals Transferred Learned Behavior From Free Flight to the Virtual Environment

**Figure 3:**
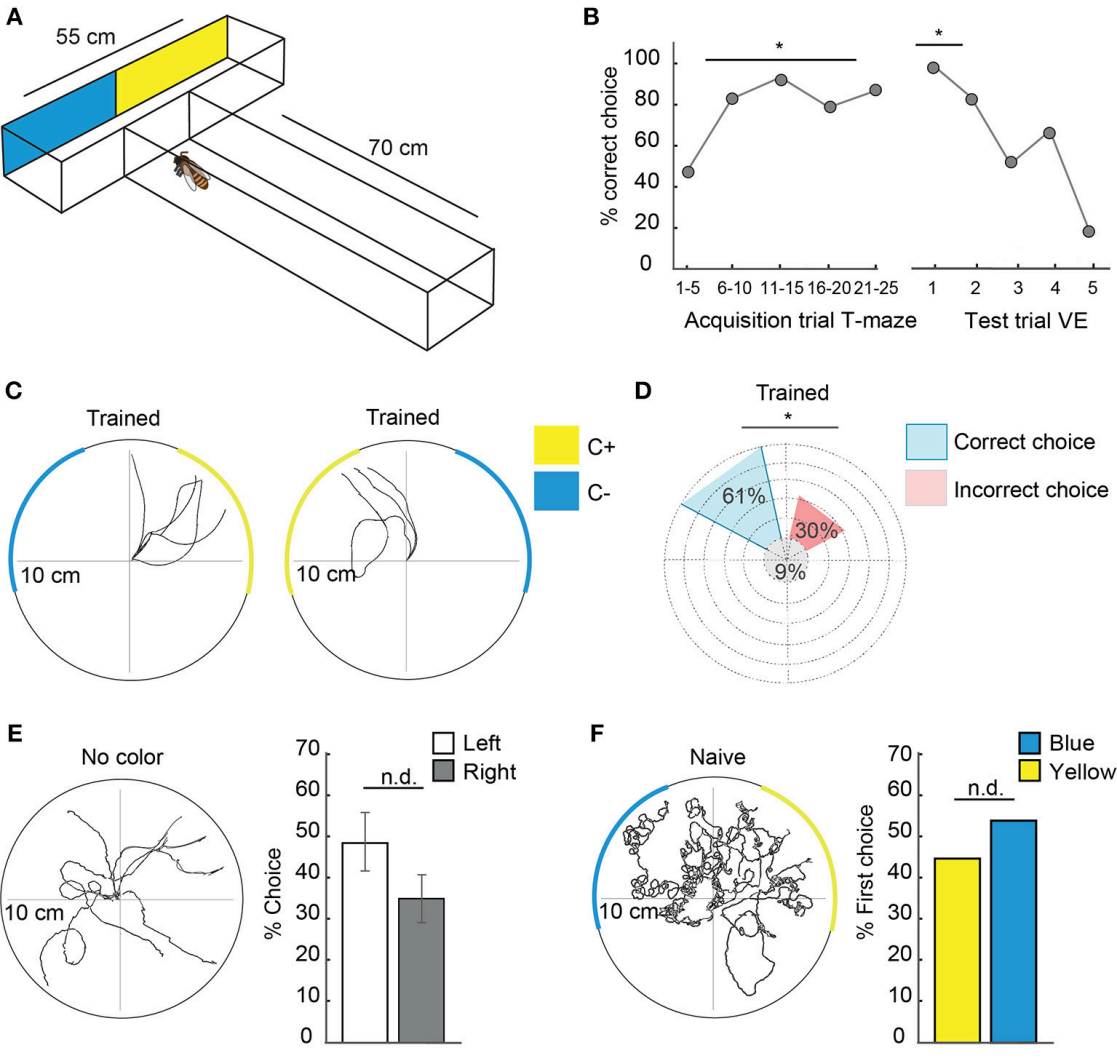
Honeybees transferred learned information from free flight experiments to the virtual environment. **(A)** T-maze experimental set-up for free-flying honeybees. The T-maze consisted of a 70 cm long tunnel, 5 cm in width and 4 cm high with a 55 cm long head side. At the end of the legs, one of the colors (yellow or blue) was rewarded with sucrose whereas the other was punished with potassium chloride solution. Bees flew toward the entrance of the T-maze, walked until the point of decision and decided for one side. Afterwards they flew back to their hive or in case of a wrong decision could enter again. Sides were switched in a pseudo-random fashion to avoid side preferences. After acquisition they were transferred to the VE and were tested in a scenario similar to the T-maze without reward (see also Figure 2A). **(B)** Left side: Acquisition curve of correct choices (*N* = 6 animals) in the T-maze with five trials depicted together from trial 1 to 25. 50% of correct choice equals chance. Right side: extinction curve of choices in the VE with the first five tests in the VE. In the VE, the same bees as in A (*N* = 6 animals) were faced with a similar situation to the T-maze and could choose one or the other color by walking toward it. Arriving at one color (10 cm distance) was counted as a decision. Afterwards the scenario was set back to CC and they could choose again. No reward was presented during the test. Sides were changed to avoid side preference dependent choices. In the first trial, all bees chose the previously rewarded color over the unrewarded. The first test trial differed significantly from chance (binomial test *p* = 0.015). **(C)**: Walking trajectories in the VE during representative test situations in a bee trained to yellow in the T-maze. Reaching the rewarded color was counted as correct choice in **(B)**. After 30 s, the colors switched sides. **(D)** Percentage of correct and incorrect choices in the VE with trained colors (yellow or blue) after acquisition in the T-maze pooled for all bees (*N* = 6 animals, *n* = 150 choices). Significantly more choices were delivered to the previous C+ over the C– (binomial test *p* = 0.0001). **(E)** Walking trajectories of a bee trained in the T-maze in a VE scenario without colored stripes but with skyline and checkerboard. Comparison of percentage choices for left and right in the VE shows no significant side differences (*N* = 6, *n* = 114 choices, Two one-sided *t*-test, *p* = 0.03). **(F)** Walking trajectories of naïve bees in a scenario with colored stripes, skyline, and checkerboard. No preferences for one of the colors was found (Two one-sided *t*-test, *p* = 0.018, *N* = 12 animals, *n* = 12 choices). VE, virtual environment. Asterisks indiacte statistical significance.

To analyze whether animals perceived the visual stimuli in the VE and recognized them as meaningful stimuli, we performed a transfer experiment. Free-flying bees were trained to enter a T-maze (Figure 3A) in which they ran toward a T-junction with two differently colored legs. Approaching one color was rewarded with sucrose and approaching the other color was punished with potassium chloride solution. After leaving the T-maze, they flew back to their hive or -in case of a wrong decision- could enter again. To avoid side preferences, we switched the sides of reward and no-reward in a pseudo-random fashion. Subsequently the animals were transferred to the VE and tested in a scenario similar to that in the T-maze (Video S1). Here, no reward was presented. The acquisition curve of correct choices in the T-maze shows that the honeybees learned to make the correct turn toward the rewarded color (C+) and discriminated it from the unrewarded color (C–, Figure 3B, from trial 5 onwards: binomial test *p* < 0.01). After the transfer into the VE, the same bees were faced with a similar situation as in the T-maze (Figure 3A). They could choose between the two colors that they had discriminated in the T-maze by walking toward one of them. At the starting point of the test two colored stripes were presented, similar to the point of decision in the T-maze experiments. Here and in the following experiments this stimulus situation is called color choice (CC). When the animal walked toward one stripe, the stripe moved toward it by increasing its visual angle in front of it simulating the object to get closer. When the animal reached it, the color covered 180° of the screen. Arriving at one color (10 cm walking distance) was counted as a decision. Afterwards the scenario was set back to CC and the animal could choose again (Figure 3C). To avoid side preferences, the sides of the colors were changed every 30 s. Each animal (*N* = 6) was tested for 5 min. In the first trial, all bees chose the previous C+ over the C– (Figure 3B, binomial test *p* = 0.015). Significantly more decisions were made for C+ in all decisions (Figure 3D, *N* = 6 animals, *n* = 150 choices, binomial test *p* = 0.0001). A comparison of the percentage of choices demonstrates that there was no side preferences for left or right in the VE without colored stripes present [Figure 3E, *N* = 6 animals, *n* = 114 choices, two one-sided *t*-test (TOST), *p* = 0.03, TOST test analyzes data for equivalence (Walker and Nowacki, 2011)]. Naïve bees introduced into the VE for the first time showed no difference in choosing between blue and yellow (Figure 3F, *N* = 12 animals, *n* = 12 choices, TOST test, *p* = 0.018).

### Neuronal Recordings During Learning in the Virtual Environment

**Figure 4:**
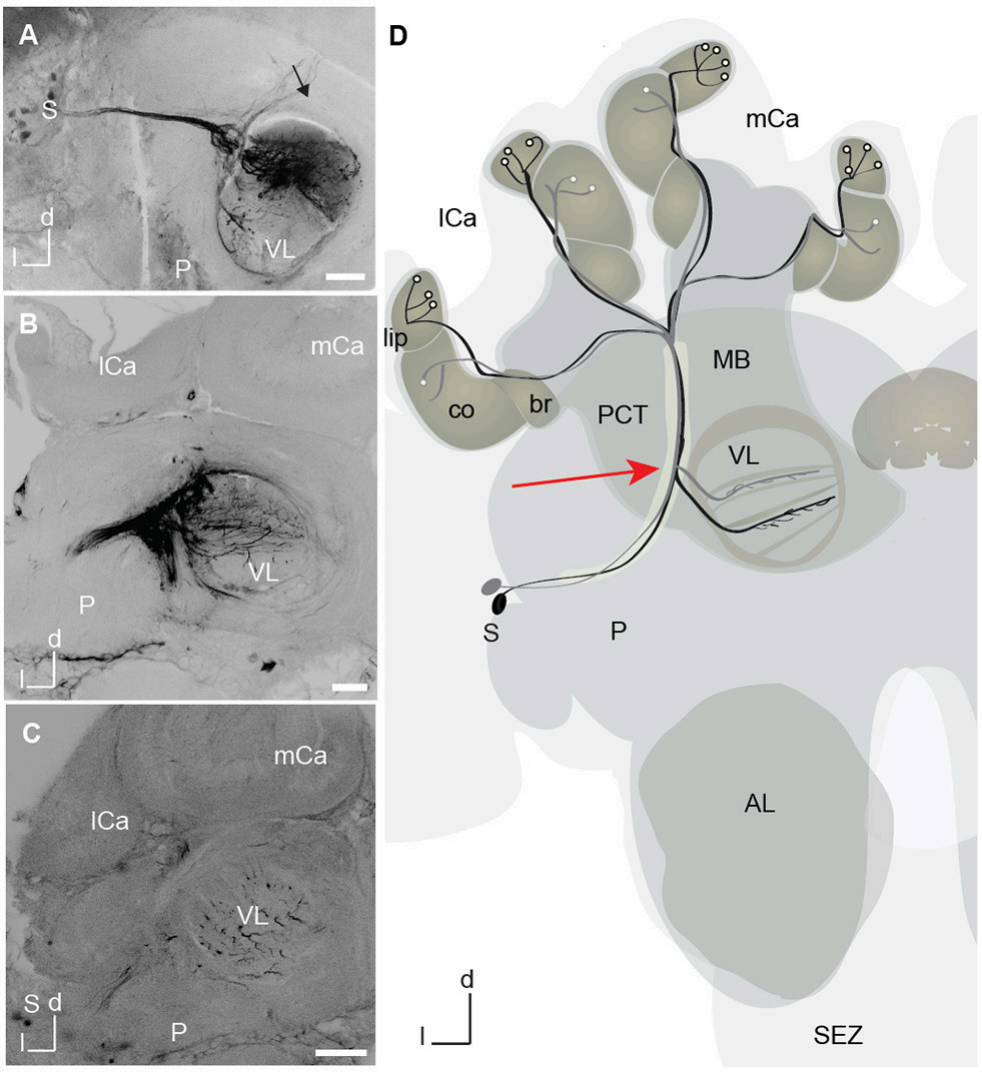
A3 mushroom body extrinsic neurons marked with dye at the tip of the recording electrode. **(A)** Projection view of a mass-fill of A3 dorsal neurons after electrode injection with Microruby on the tip of the electrode. Tracts of the dorsal somata cluster and the horizontal layers in the vertical lobe (VL) are visible. Axons project toward the calyx in the protocerebral calycal tract (PCT, black arrow) revealing A3 neurons. **(B)** Projection view of a different mass-fill of A3 neurons after electrode injection with Microruby on the tip of the electrode. Again, tracts of A3 dorsal (d) neurons are visible together with a horizontal layer in the VL. **(C)** Projection view of a mass fill of A3d neurons with arborizations in the VL. **(D)** Schematic drawing of two different innervation types of A3 feedback neurons. In black A3 neuron connecting lip and corresponding layer in the VL, in gray A3 connecting collar and corresponding layer in the VL. A3 neurons were also found to connect the basal ring and the corresponding basal ring layer in the VL. All types connect to the medial lobe. Another class-A3 lobe connecting neurons—do not innervate the calyx (not shown) (Zwaka et al., 2018). The red arrow points to the recording site. AL, antennal lobes; br, basal ring; CB, central body; co, collar; d, dorsal; P, protocerebrum; l, lateral; lCa, lateral calyx; MB, mushroom bodies; mCa, medial calyx; PCT, proto cerebral calycal tract; SEZ, subesophageal zone; S, Soma; VL, vertical lobe.

The search for neural correlates of learning in a VE requires the combination of stable recording from central neurons and free movement of an animal. We performed 24-h lasting extracellular recordings from the area where A3 mushroom body extrinsic neurons bifurcate (Figure 4). The recording site could be well selected visually by placing the recording electrodes at the lateral rim of the vertical lobe (red arrow in Figure 4D). After the experiment, the recording site was verified by dye marking via the tip of the recording electrode (Figures 4A–C).

**Figure 5:**
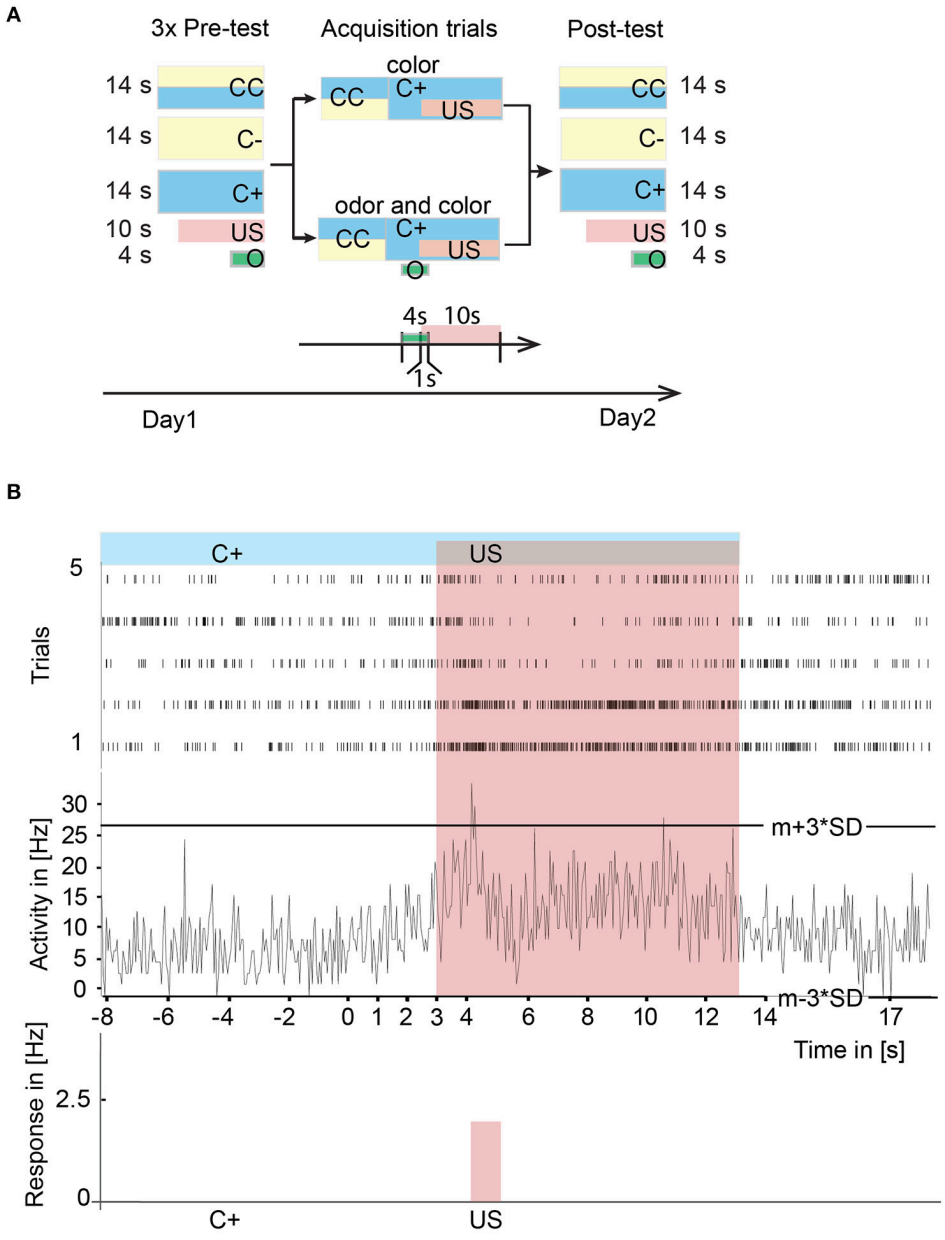
Experimental paradigms and neural correlates of A3 neurons of stimuli during learning in the virtual environment. **(A)** Experimental design for operant learning in the VE. On the first day in a pre-test, we presented a scenario with two colored stripes (CC) and two scenarios with only one color (C+, C–), each three times in a pseudo-random fashion for 14 s with an inter trial interval of 1 min. Additionally, we presented the sucrose reward (US) three times alone. In experiments with odor presentation in the VE, we additionally tested an odor three times for 4 s in the pre-test. During acquisition, the animal could walk freely in the VE with two stripes (CC) one of which (C+) was rewarded and one was unrewarded (C–). After reaching C+, in the color-only paradigm the US was presented to the animal for 10 s. Afterwards, the C+ was switched off simultaneously with the US. In the color-odor paradigm, a 4 s lasting odor and subsequently the US was presented after reaching C+. In the post-test situation on the day after the acquisition, CC, C+, C–, the US, and the odor were presented to the honeybee again. **(B)** Peri-stimulus time histogram over five acquisition trials of single unit activity in the upper panel and mean trial activity in the middle panel. Activity exceeding a three-standard deviation plus mean threshold (m+3^*^SD) or falling below m-3^*^SD threshold was noted as a response to the stimulus. The sum of the responses was divided by the duration of the stimulus and noted as a significant response in Hz (lower panel). Here, a significant increase in response to the US has been detected. CC, rewarded and unrewarded colored stripes; C+, rewarded color; C–, unrewarded color; O, conditioned stimulus (odor); US, unconditioned stimulus (reward); VE, virtual environment.

To study operant learning in the VE we allowed the animal to walk freely through the VE. Two different training paradigms were used in the VE: Either only a color was rewarded (color-only) or the rewarded color was paired with an odor (color-odor, Figure 5A). Each bee was trained only in one paradigm. At the beginning of each acquisition trial, two colored stripes were presented and the bee walked toward one of the two colors such that it reached a particular size and location in front of the animal. This size and location equaled 10 cm walking distance from the starting point toward the center of the stripe thus covering 180° of the screen (rewarding site). After arriving at a previously determined color (blue or yellow, randomly selected by the experimenter), the animal was rewarded with 30% sucrose solution (rewarded color, C+). When this situation was reached a rewarding device turned toward the animal, and the animal could lick the sucrose solution for 10 s. Sides were switched to distinguish between side and color effects on unit activity.

Single unit activity was analyzed (for sorting procedure see Figure 1) in peri-stimulus time histograms with a bin size of 50 ms in all trials (Figure 5B). Activity exceeding the mean plus three standard deviations (from activity before stimulus onset) threshold (m+3^*^SD) or falling below m−3^*^SD threshold was noted as an excitatory or inhibitory response to the stimulus, respectively. The response strength in Hz to the stimulus was calculated with the sum of the unit's activity divided by the duration of the particular stimulus in seconds.

We performed pre-tests to get the neural responses of the respective naïve animal toward the stimuli that were tested in a pseudo-random fashion before operant training started: (1) both a blue and a yellow stripe (CC) for 14 s. In this moment, the bee could walk toward the C+ or C– (called CC for color choice), (2) C+ (later the to be rewarded color) alone for 14 s, (3) C– (later the not to be rewarded color) alone for 14 s, (4) US three times for 10 s each. (5) In the color-odor paradigm, we additionally tested the odor three times in the pre-test for 4 s.

In a memory test on the day after acquisition (post-test), all color stimuli (CC, C+, and C–), the US, and the odor were presented again, separately. This remained the only extinction tests in the experiment.

Comparing all bees, there were only two points in time that were fixed: The time the color was switched on and the time the reward was delivered. Therefore, we analyzed the neural activity when C+, C–, or CC was switched on, during the time the animal had arrived at C+ shortly before the reward (US) was switched on, and during the US. We compared the activity of the single units to the stimuli prior to acquisition (pre-test), during acquisition (acquisition) and in a memory test after acquisition (post-test) without a reward. No significant inhibitory responses were determined, most likely due to the rather low spike activity and the high criterion for the significant test (mean minus three standard deviation). Only animals that received all pre-tests, acquisition trials and post-test with a stable extracellular recording over two consecutive days were analyzed (*N* = 29 Units).

**Figure 6:**
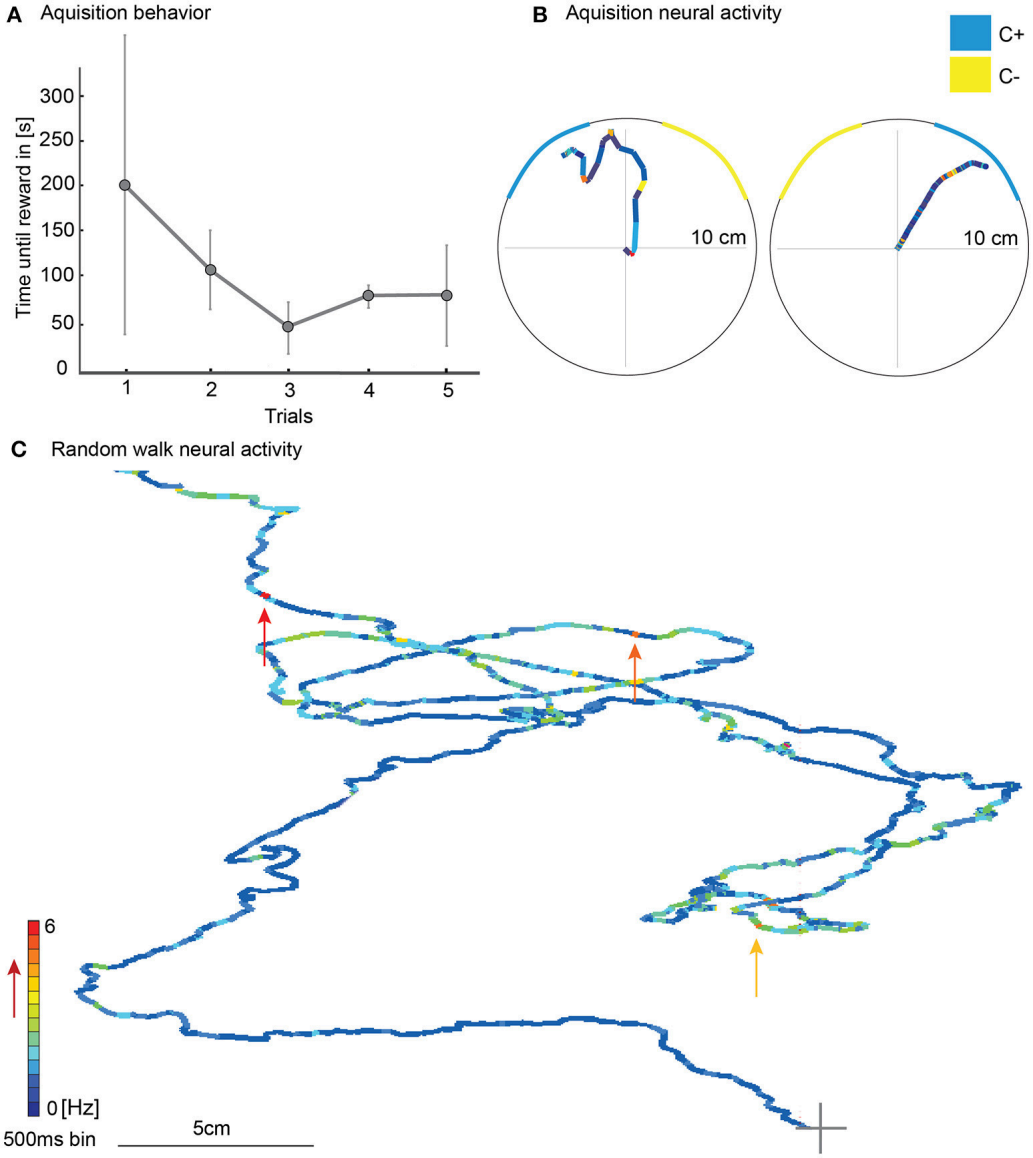
Walking behavior in the virtual environment during learning with simultaneously recorded single unit activity of a mushroom body extrinsic neuron. **(A)** The time until the animal reached the reward decreased during the five acquisition trials although not statistically significantly [rmANOVA, *N* = 3, *F*_(4, 8)_ = 0.61, *p* = 0.66]. **(B)** Here the animal walked toward the blue stripe and was rewarded when it reached the rewarding site. The neuronal activity of one responding unit is plotted in false color on top of the walking trajectory. The duration of one colored bin equals 500 ms. The warmer the color, the higher the activity of the unit (see false color scale in **C**). In two approaches, the activity changed when the animal walked toward the blue stripe shortly before it was rewarded. **(C)** Example for one single unit activity in Hz plotted on the walking trajectory in a scenario without colored stripes. Single unit activity varied over the course of the walk without any indication of site or place preference. Arrows indicate unit activity at points of high activity. A gray cross marks beginning of walking trace; Vertical bars indicate standard deviation; Hz, Hertz.

During the five acquisition trials of the color-only training, the mean of the time until the animals reached the reward decreased although not significantly [Figure 6A, *N* = 3; rmANOVA, *N* = 3, *F*_(4, 8)_ = 0.61, *p* = 0.66]. Walking traces from acquisition trials with corresponding unit activity showed changes in neural activity (Figure 6B). During random walk without any colored stripes in the VE, the single unit activity varied over the course of the walk and showed no indication of a place preference (Figure 6C).

### Response Rate Changes to the Rewarded Stimuli During Learning

#### Color-Only Experiments

**Figure 7:**
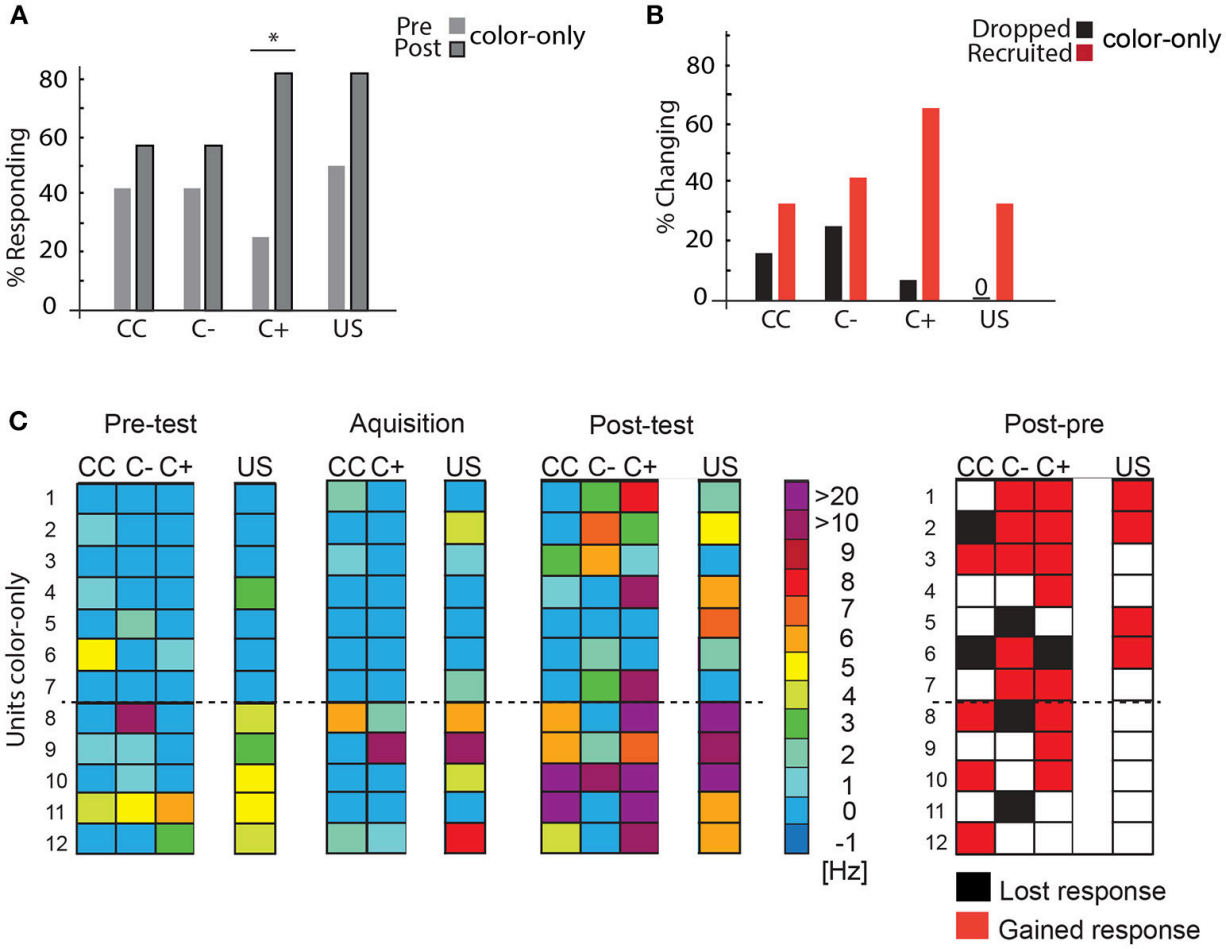
Learning-related plasticity during the color-only paradigm. **(A)** Percentage of responding units to the C+ increased significantly from the pre-test to the post-test (McNemar test, *p* = 0.04). **(B)** Percentage of changing responses categorized as being recruited and dropping out as measured for the different stimulus conditions. **(C)** Left side: Single unit responses in the pre-test, during acquisition and in the post-test for the different stimulus conditions. Excitatory responses are marked in warm colors. The warmer the color, the higher the change in the respective activity. No change in activity compared to before is marked in blue. No inhibitory responses were found during the analysis most likely due to the rather low spike activity and the high criterion for the significant test (mean minus three standard deviation) (*N* = 12). The single unit activity changed from the pre-test to the post-test and during the acquisition. Right side: Summary of recruited or dropped units after learning. Here, dropped and recruited refers to post-test compared to the pre-test for all stimuli. Black indicates that the unit responded less to the respective stimulus in the post-test than in the pre-test, red indicates that the unit responded more than in the pre-test, white indicates no change; US, unconditioned stimulus (reward). CC, response during the color choice with both colors blue and yellow present; C+, response to the rewarded color; C–, response to the unrewarded color; US, response to the sucrose reward. Asterisks indiacte statistical significance.

Twelve units were recorded while the bees were trained in the VE to approach one of two colors (blue or yellow) that was subsequently rewarded. Almost all units changed their responses during the experiment with both an increase in the number of responding units and an increase in the spike frequency to C+ (Figure 7). First, we analyzed the changes in the number of units responding to the stimuli during the pre-test and the post-test and found higher response activity for the C+ (Figure 7A, McNemar test, *p* = 0.04). Such a global analysis may, however, not uncover learning-related plasticity in single units due to opposing changes, e.g., units being recruited or units dropping out. Recruitment in this case relates to a neuron, which had not responded to the stimulus before and started to respond after acquisition or in the test (after memory formation). Likewise, a neuron that responded to a stimulus before acquisition and did not respond in the acquisition or post-test situation anymore is regarded as dropping out. An analysis revealed that single units were recruited and dropped (Figure 7B).

When we compared responses to all stimuli (CC, C+, and C–) during pre-test and post-test (Figure 7C), we found that in sum 10 out of 12 units (Units 1, 2, 3, 4, 6, 7, 8, 9, 10, 12) respond to at least one more stimulus (CC, C+, and C–) after learning in the post-test than in the pre-test. One unit gained a response to the US (Unit 5).

#### Color-Odor Experiments

Sixteen units were recorded while the bees were trained in the VE to approach one of two colors (blue or yellow) that was subsequently paired with an odor and the reward (compare Figure 5). As in the previous experiment, we compared the activity of the single units to the stimuli prior to acquisition (pre-test), during acquisition (acquisition) and in a memory test after acquisition on the next day (post-test) without a reward. In these color-odor experiments, we additionally analyzed the activity during the odor presentation. We hypothesize that the odor stimulus preceding the reward may become salient and thus indicative in uncovering learning-related plasticity.

**Figure 8:**
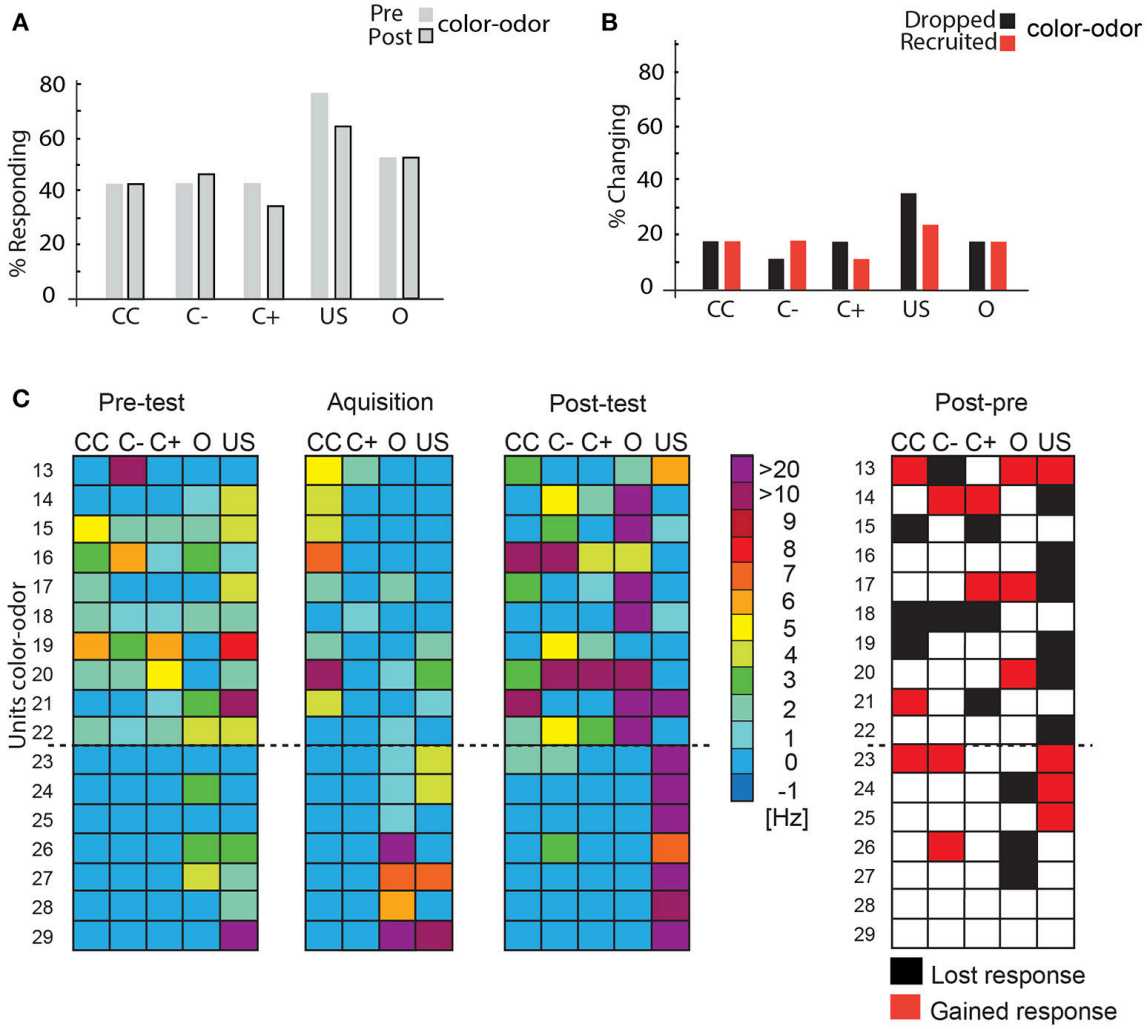
Learning-related plasticity during the color-odor paradigm. **(A)** No significant changes occurred in the percentage of responding units from the pre- test to the post-test (McNemar test, *p* >0.5). **(B)** Percentage of changing responses categorized as being recruited and dropping out as measured for the different stimulus conditions. **(C)** Left side: Single unit response in the pre-test, during the acquisition and in the post-test for the different stimulus conditions. Excitatory responses are marked in warm colors. The warmer the color, the higher the change in the respective activity. No change in activity compared to before is marked in blue. No inhibitory responses were found during the analysis most likely due to the rather low spike activity and the high criterion for the significant test (mean minus three standard deviation) *N* = 17. Right side: Summary of recruited or dropped units after learning. Here, dropped and recruited refers to post-test compared to the pre-test for all stimuli. Black indicates that the unit responded less to the respective stimulus in the post-test than in the pre-test, red indicates that the unit responded more than in the pre-test, white indicates no change; US, unconditioned stimulus (reward). Only two units did not change their responses (number 28 and 29). CC, response during the color choice with both colors blue and yellow present; C+, response to the rewarded color; C–, response to the unrewarded color; US, response to the sucrose reward.

When we compared the pre-test to the post-test no significant changes were found in the percentage of responding units (McNemar test > 0.05, Figure 8A). However, analyzing the percentage of units that changed their responses to the stimuli revealed that an equal number of units was recruited and dropped out. More units were recruited than dropped during C– stimulation. These changes are not significant (Figure 8B, McNemar test *p* > 0.05). During acquisition, only two units (Units 13, 18) responded to the C+. Comparing responses to stimuli during post-test and pre-test (Figure 8C) showed that nine units (Units 13, 14, 17, 20, 21, 23, 24, 25, 26) responded to at least one more stimulus in the post-test than in the pre-test, whereas six lowered their response activity to stimuli (Units 15, 16, 18, 19, 22, 27). Two units did not change their responses (Units 28 and 29).

### Spike Rates Change of Units During Learning

After analyzing the changes in the absolute number of units responding to the stimuli, we next analyzed the changes in spike firing rates (SFR) to the respective stimuli. Even though the absolute number of units responding might not be different, this analysis helps us detect an increase or a decrease in firing rate to a stimulus. We compared the conditions during the pre-tests with those during acquisition, and during the pre-tests with those during the post-test for all tested stimuli. We calculated the ΔSFR using the changes of spike rates during stimulation as compared to the spontaneous spike rate 20 s before, including only those data in which the changes exceeded the 3^*^SD threshold in the PSTHs. The spike activity was normalized by taking the ratio between stimuli response in pre-test and training or pre-training and test (see Experimental Procedures). Thus, ΔSFR defines the change in spike firing rate from e.g., pre-test to test toward a stimulus divided by total response. A positive ΔSFR indicates an increase in firing rate, a negative ΔSFR a decrease, and ΔSFR of 0 indicates no change (Hussaini and Menzel, 2013). ΔSFRs of all units were analyzed together applying the Wilcoxon signed-rank test against a hypothetical change of zero. Again, we shall deal with the color-only paradigm first and then with the color-odor paradigm.

**Figure 9:**
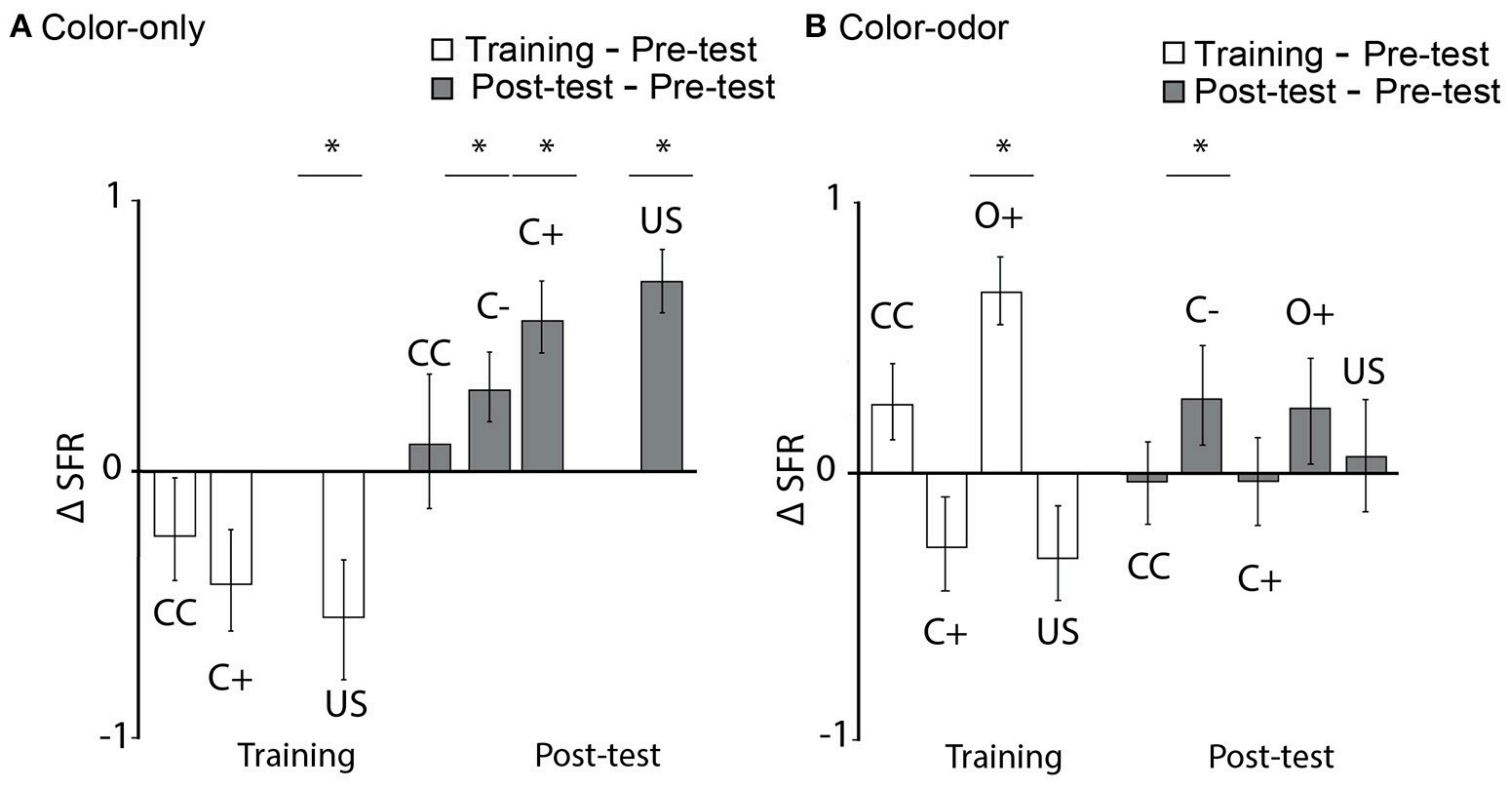
Learning-related changes in the spike firing rate. **(A)** Color-only paradigm: Normalized firing rate (ΔSFR) decreased significantly for the US during training (training as compared to pre-test, Wilcoxon signed-rank test, *N* = 12, US: *p* = 0.04) and increased significantly for C–, C+ and US in the post-test as compared to the pre-test (Wilcoxon signed-rank test, *N* = 12, C–: *p* = 0.02, C+; *p* = 0.04, US: *p* < 0.0001). **(B)** Color-odor paradigm: ΔSFR increased significantly during training for the rewarded odor (O+) (Wilcoxon signed-rank test, *N* = 17, O+: *p* < 0.00046), and increased significantly for C– in the post-test as compared to the pre-test (Wilcoxon signed-rank test, C–: *p* = 0.016). Asterisks indiacte statistical significance.

Firing rate decreased significantly for US during color-only training (Figure 9A, training as compared to pre-test, Wilcoxon signed-rank test, *N* = 12, US: *p* = 0.04) but increased during the test (Figure 9A, Wilcoxon signed-rank test, *N* = 12, *p* < 0.0001). Similarly firing rate increased significantly to both the rewarded and unrewarded color (Figure 9B, Wilcoxon signed-rank test, *N* = 12, C–: *p* = 0.02, C+; *p* = 0.04). A significant increase was found for the rewarded odor (Wilcoxon signed-rank test, *N* = 17, O+: *p* < 0.00046) during color-odor training but not for the rewarded color (C+). The post-tests revealed a significant increase in firing rate for the non-rewarded color C– (Wilcoxon signed-rank test, *N* = 17, C–: *p* = 0.016). Again, a very different pattern of learning-related plasticity was found between the two paradigms, color-only and color-odor. These results indicate changes in spike firing to the stimuli in the range of short-term memory (training) but also changes in neuronal activity that persist until the next day (post-test).

### Temporal Dynamics of Spiking Rate Changes During Learning

We found learning-related spike rate changes for several stimuli, but not for the CC situation. Animals saw both colors appearing at the same time and walked toward one or the other color during the CC scenario in order to get a reward (compare Figure 2A). It seems surprising that no neural response changes were observed during the CC condition. To detect fast spike rate changes that are not detected by our global analyses during stimulation, we compared the time courses of spiking by inter-spike interval (ISI) analysis during pre-test, acquisition, and post-test, and focused on the time window directly after stimulus onset (600 ms). This method detects subtle excitatory or inhibitory rate changes that extend over several 100 ms during stimulation (Strube-Bloss et al., 2011). Additionally, we analyzed the ISI that occurred during acquisition by comparing median ISI during onset of C+ to median ISI during US onset. The medians of the pre-test- and the post-test- ISI was extracted and statistically analyzed for every unit using a Wilcoxon signed-rank test (see also Figure 1 for median ISI). We subtracted the median ISI in the post-test from the median ISI in the pre-test for every unit separately for depicting the changes.

**Figure 10:**
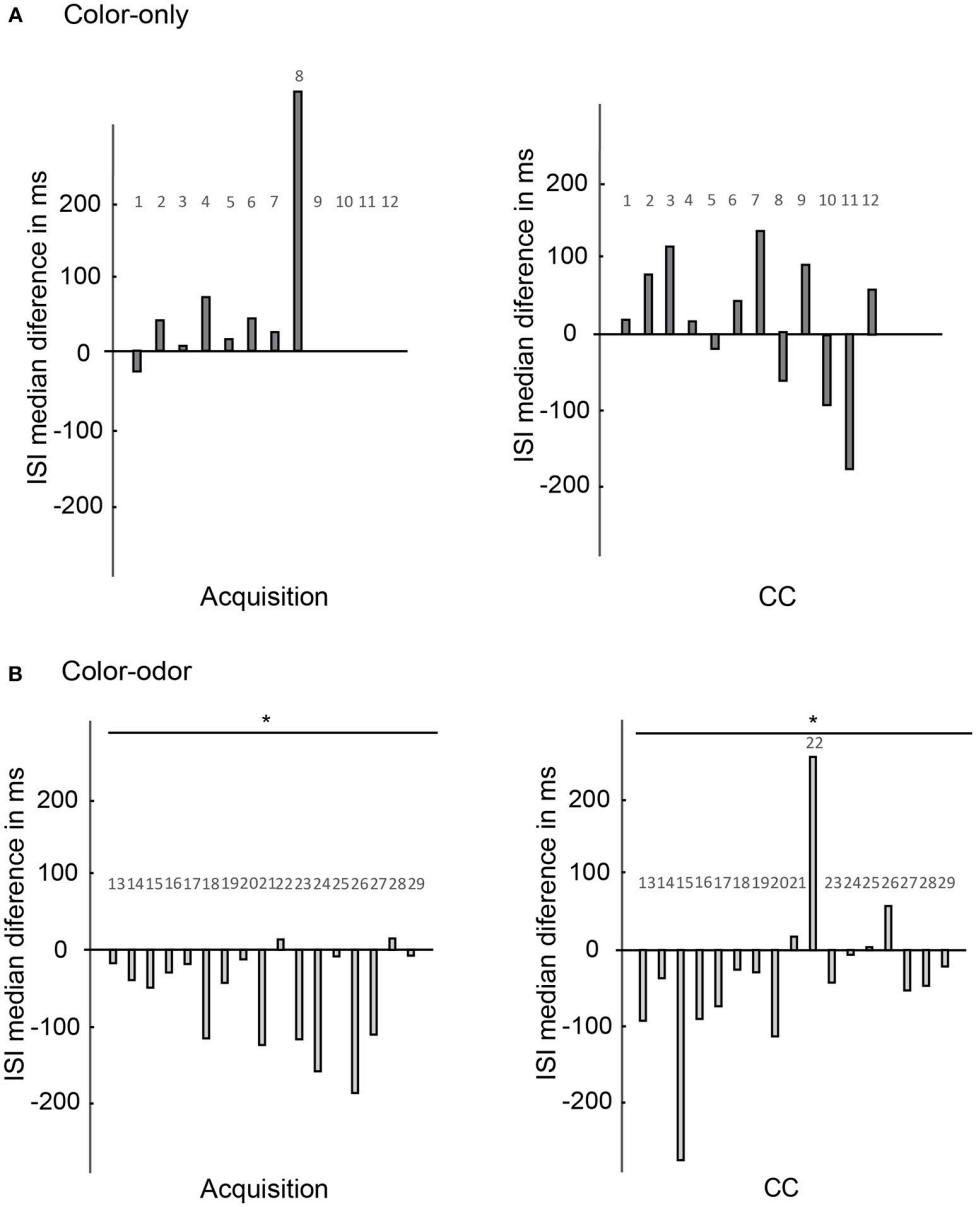
Changes of the ISI medians as compared for the acquisition and the CC phase. Changes of ISI medians of the units (x-axis) are calculated for the 600 ms after stimulus onset in the pre-test and in the post-test (y-axis). **(A)** Color-only paradigm: ISI medians of 12 units (x-axis) during the acquisition phase and the CC phase. The changes of ISI medians are not significantly different for color onset as compared to reward onset (*N* = 12, Wilcoxon signed-rank test, *p* > 0.05). No significant changes are found for the CC phase comparing the pre-test and post-test (*N* = 12, Wilcoxon signed-rank test, *p* > 0.05). **(B)** Color-odor paradigm: Changes in ISI medians are significantly different during the acquisition for the color and the reward onset (*N* = 17, Wilcoxon signed-rank test, *p* = 0.005). The changes of ISI medians are also significantly different for the CC for the pre-test and post-test comparison (*N* = 17, Wilcoxon signed-rank test, *p* = 0.046). Asterisks indiacte statistical significance.

No significant differences in the changes of ISI medians were found for any tested conditions for animals trained in the color-only paradigm (Figure 10A, *N* = 12, Wilcoxon signed-rank test, *p* > 0.05).

However, significant differences appeared for the CC situation in animals trained in the color-odor paradigm (Figure 10B right side, Wilcoxon signed-rank test, *N* = 17, *p* = 0.046). The changes in ISI medians indicate that learning lead to higher spike frequencies in the CC test conditions immediately after stimulus onset. Furthermore, the ISI medians became significantly shorter at color onset as compared to sugar onset during acquisition (Figure 10B left side, Wilcoxon signed-rank test, *p* = 0.005). The ISI medians did not change significantly from pre-test to post-test for the C+ and C–, the odor and the US (data not shown, Wilcoxon signed-rank test *p* > 0.05). As a control we analyzed the ISI over the whole time of the experiment. ISI did also not change significantly when we compared the ISI of the time before the pre-test without stimulation with the ISI of the time before the post-test without stimulation (Wilcoxon signed-rank test *p* > 0.05).

These results show that even though no changes for the CC were found when comparing the spike rate changes over the whole stimulus (Figure 9), fast changes indeed occurred within 600 ms after stimulus onset in one of our paradigms, the color-odor training but not the color-only training marking the operant conditions of this experimental conditions.

## Discussion

We developed a virtual environment (VE) for honeybees that allowed us to combine stable recordings from mushroom body (MB) extrinsic neurons with a learning assay. The VE is suitable for testing bees both after operant color learning of free-flying bees, and de-novo color-only or color-odor training inside the VE. MB extrinsic neurons (ENs) changed their responses both to the rewarded color (C+) and unrewarded the color (C–). For the first time, we found evidence for an involvement of A3 neurons in operant learning in the honeybee.

In operant learning, animals establish an association between an external stimulus and their behavioral responses by trial and error. This form of learning is also called instrumental, because the animal's own behavior is instrumental to obtaining some outcome (Wynne and Udell, 2013). The requirement is an external stimulus that is only present if the animal performs a certain behavior. This stimulus can be positive or negative and subsequently alters the associated behavior (Mackintosh, 1974; Spencer et al., 1999). A VE provides us with the opportunity to study operant learning while the corresponding brain activity is monitored. Although operant learning in freely moving invertebrates has been widely studied (Giurfa and Menzel, 2013; Perry et al., 2013; Hawkins and Byrne, 2015) the underlying neural mechanisms are still largely unknown. Evidence for neural correlates of operant learning in invertebrates comes from *Drosophila, Aplysia* and *Lymnae*. These studies suggest that different cellular and network mechanisms underlie classical and operant conditioning (Brembs et al., 2002; Hawkins et al., 2006; Hawkins and Byrne, 2015). In the honeybee, mushroom body extrinsic neurons (MB ENs) were extensively studied in classical conditioning paradigms and found to change their response properties during olfactory conditioning (Mauelshagen, 1993; Grünewald, 1999; Okada et al., 2007; Haehnel and Menzel, 2010; Strube-Bloss et al., 2011; Hussaini and Menzel, 2013; Menzel, 2014; Filla and Menzel, 2015). A3 MB ENs are particularly interesting in this context because it is known that they change their responses during context-dependent classical conditioning with learning-related neural changes both for the visual context and the olfactory cue (Filla and Menzel, 2015). These data suggested a multisensory and value-integrating pathway specifically designed for action selection comparable to neurons in the mammalian prefrontal cortex.

We applied two different learning paradigms where the active selection and approach had to be performed for either a colored target (color-only) or for a color that was subsequently combined with an odor (color-odor). It is known from behavioral studies with freely flying bees and bees conditioned to the proboscis extension response that odors are particularly salient stimuli (Menzel, 1990). In our context, studies in *Drosophila* are notable documenting an involvement of the mushroom bodies in complex stimulus learning and decision making (Zhang et al., 2007; Xi et al., 2008; Yi et al., 2013; Solanki et al., 2015).

Neural activity in the color-only paradigm changed significantly for the rewarded color C+ (Figure 7A). About 70% of the units were recruited and only few stopped responding to the C+. In response to the unrewarded color C– units were recruited and dropped to a more equal amount (Figure 7B). These findings resemble those found in other MB ENs (A1/A2) during differential olfactory classical conditioning (Strube-Bloss et al., 2011). Resembling classical conditioning, however might be explained due to the fact that our operant conditioning paradigm is not purely operant as possible for example in Drosophila. These results corroborate the conclusion that MB ENs appear to code learning-related plasticity for the CS+ by a dominance of response increase. A different strategy might be involved in coding responses to the unrewarded stimulus CS- by keeping the overall excitation level rather constant increasing and decreasing the neural responses in participating neurons.

Response changes in the color-odor paradigm differed partly from those found in differential olfactory classical conditioning mentioned above. In a classical conditioning experiment where a color context announced the rewarded odor cue, the number of A3 neurons and their discharges increased for the odor cue (Filla and Menzel, 2015). In our learning paradigm however, the number of units responding to the C+ did not change significantly (Figure 8A), and a similar number of units were recruited and dropped out for all stimuli (Figure 8B). However, during training, we observed a significant enhanced spike rate to the rewarded odor (Figure 9B) and a tendency for a reduced rate to the color during training. This observation fits previously reported changes in the PE1 neuron (Okada et al., 2007; Hussaini and Menzel, 2013). This single identified MB EN, decreased its response to a classically conditioned odor (Okada et al., 2007; Hussaini and Menzel, 2013) and increased it to a rewarded context (Hussaini and Menzel, 2013). As this cell is thought to get inhibitory input from PCT (or A3) cells (Rybak and Menzel, 1998), its response changes fit very well to our data.

Differences between classical conditioning and operant learning can also be found in other experiments with MB ENs. A1/A2 ENs, for example, develop their learning-related spike rate changes during the 3 h after classical conditioning (Strube-Bloss et al., 2011). We found such changes in what are most likely A3 neurons, already during the training process (Figure 10). We conclude from our verified recording site (see Experimental Procedures) and the physiological properties of the recorded cells that the analyzed spikes are A3 neurons, at least to a large part. In our experiments 25 out of 29 units changed their spiking frequency before training toward the presentation of an odor, a color or the sucrose reward. A3 neurons typically react with an increase toward a sucrose stimulation (Grünewald, 1999) and a change in the spiking activity toward odors and colors (Grünewald, 1999; Filla and Menzel, 2015).

In the search for neural correlates of operant learning, one would like to analyze the point of decision where the animal initiates a walk toward one of the two colors. Unfortunately, such decision points were not obvious since every walking trajectory included several turns or stops making it impossible to isolate attempts to walk toward the color. Thus, we decided to examine fixed points in every acquisition trial for all bees and selected two specific moments in which the scenario with both colors showed up (CC). This is the starting point for each acquisition trial. Therefore, one might expect changes in neural responses at this point when the animal responds behaviorally and starts to walk toward the C+ to get the reward. Indeed, in the color-odor paradigm, the inter-spike interval (ISI) during the CC was significantly lowered after training (Figure 10B). Filla and Menzel (2015) showed that after visual pre-training the presence of a visual context induces an attention effect, reflected in faster response to the reward odor than testing the reward odor alone. The increased ISI to CC might reflect such an attentional effect. After the onset of the CC a behavioral response from the animal is needed to gain the reward.

In summary, the VE for honeybees allowed us to search for neural correlates of learning while animals navigated in an environment that responded to their actions. We demonstrate that honeybees transfer learned information from free flight learning experiments to the VE, documenting its suitability for testing operant behavior. Previously, this transfer has been reported to be unpredictable most likely due to the constrained movement and impaired active vision in the VE (Buatois et al., 2018). The VE also enabled us to study neural correlates of learning over several hours. We found significant changes in neural activity after learning to both the rewarded and unrewarded colors in the color-only paradigm. These forms of neural plasticity resemble processes found in classical conditioning experiments with honeybees. Learning-related neural plasticity differed in the color-odor paradigm from those reported in classical conditioning (Table 1). These forms of neural plasticity can be interpreted as indicating attentional effects. Our results suggest the involvement of the MB in operant learning.

**Table 1:** Comparison of changes in neural activity after learning in VE and classical conditioning.

**Experimental result color-only**	**Similar to classical conditioning**	**Different from classical conditioning**
Change in neural activity for the rewarded color C+ after conditioning. About 70% recruited units and only few that dropped out (Figures 7A,B).	Around 50% of MB ENs change their odor response after differential olfactory classical conditioning dominated by CS+ odor recruitment (Strube-Bloss et al., 2011).
Recruited and dropped out units in response to the unrewarded color C– after conditioning (Figure 7B).	Recruited and dropped units to the CS– odor after conditioning (Strube-Bloss et al., 2011).	Less responding units to the CS– odor after conditioning compared to before, more units dropped than recruited (Strube-Bloss et al., 2011).
**Experimental result color-odor**	**Similar to classical conditioning**	**Different from classical conditioning**
No significant change in the number of units responding to the C+ after conditioning (Figure 8A). Similar number of recruited and dropped out units for all stimuli (Figure 8B).	Recruited and dropped units to the CS– odor after conditioning (Strube-Bloss et al., 2011).	Increased number of A3 neurons and their discharges for the CS+ odor where a particular color context announced the particular rewarded CS+ odor (Filla and Menzel, 2015).
Significantly enhanced spike rate during training to the rewarded odor (Figure 9B) and a tendency for a reduced rate to the color during training.	PE1 neuron decreased its response to a classically conditioned odor (Okada et al., 2007; Hussaini and Menzel, 2013) and increased it to a rewarded context (Hussaini and Menzel, 2013). PE1 is thought to get inhibitory input from or A3 cells (Rybak and Menzel, 1998).	
Changes in neuron response during the training process (Figure 10B).		A1/A2 ENs develop learning-related spike rate changes after classical conditioning (Strube-Bloss et al., 2011).

## Author Contributions

HZ: conception and design, acquisition of data, analysis and interpretation, writing the article; RB: conception and design, revising the article; SL and MJ: acquisition of data; SH, JG, and RA: writing custom program for virtual environment and analysis software; SM: building virtual environment set-up; RM: conception and design, writing and revising the article; SL and MJ: acquisition and analysis of data.

### Conflict of Interest Statement

The authors declare that the research was conducted in the absence of any commercial or financial relationships that could be construed as a potential conflict of interest.

## Supplementary Material

Video S1 Video shows a honeybee walking in the virtual environment. The free-flying bee was trained in a T-maze to associate yellow with a sucrose reward and blue with a potassium chloride punishment. Subsequently the animal was transferred to the VE and tested in a scenario similar to the point of decision in the T-maze. When the animal walked toward one stripe, the stripe turned in front of it depending on the rotatory movement of the spherical treadmill. When it kept walking toward the stripe, it got bigger, simulating the object getting closer, and stopped, when it reached it. Afterwards the scenario set back to the decision point and the animal could choose again. Sides were switched in a pseudo-random fashion to avoid side preferences. The bee in the video repeatedly chooses to walk toward yellow.

Figure S1 **(A)** Example of all single unit waveforms (500 events) for one animal with their corresponding principal component analysis **(B)**. **(B)** Upper panel: Three dimensional plot of principal component analysis for all units in **(A)**. Lower panel: all units crossing minus three standard deviations lower left panel and crossing plus three standard deviations lower right panel. PCA is rotate in 3D for better display.
