# Supplementary figures and images for: Learning and Its Neural Correlates in a Virtual Environment for Honeybees
Source: Front Behav Neurosci. 2019 Jan 25;12:279. doi: 10.3389/fnbeh.2018.00279 (PMC6355692; doi:10.3389/fnbeh.2018.00279)

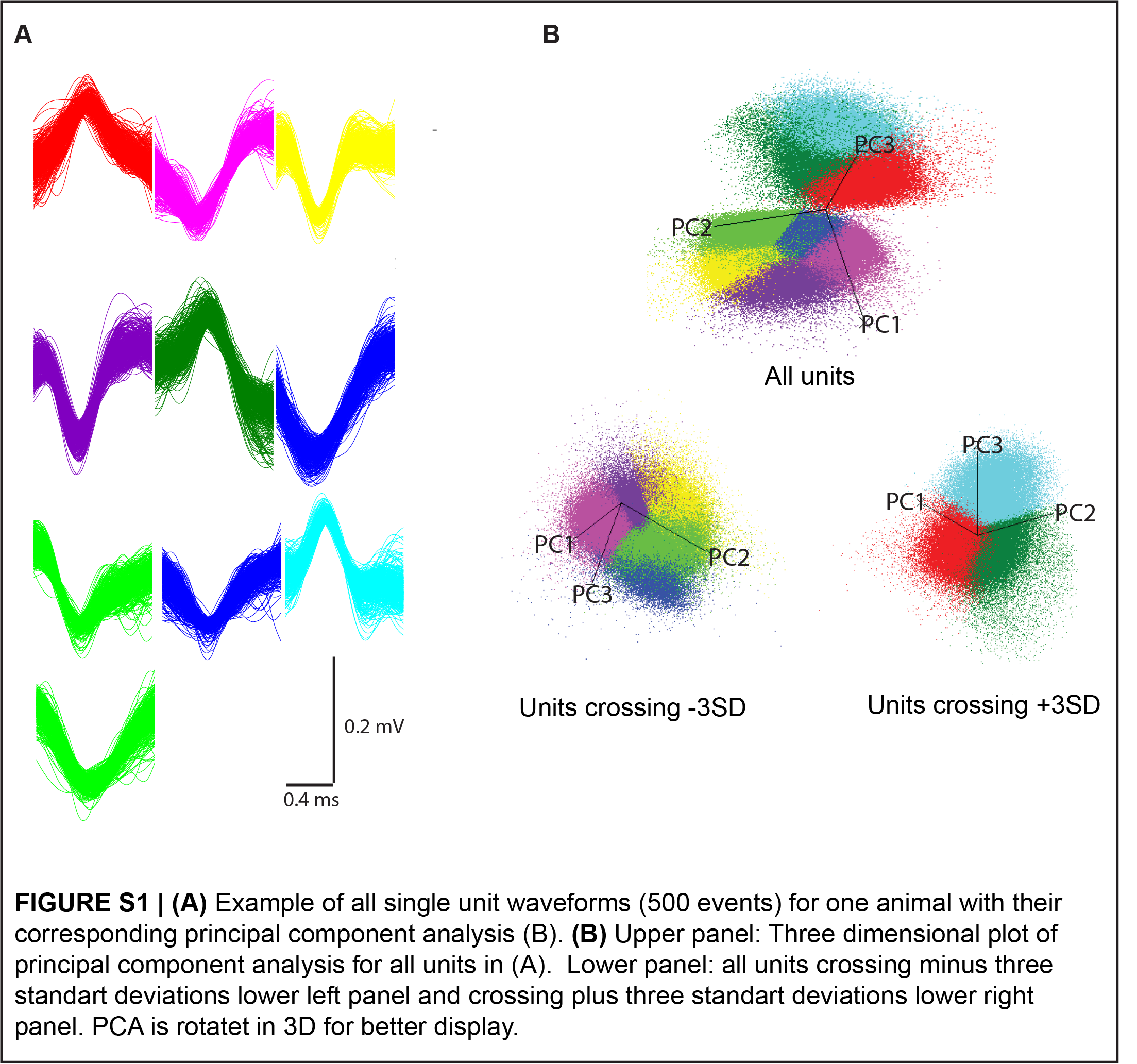

Supplement: Figure S1 — (A) Example of all single unit waveforms (500 events) for one animal with their corresponding principal component analysis (B). (B) Upper panel: Three dimensional plot of principal component analysis for all units in (A). Lower panel: all units crossing minus three standard deviations lower left panel and crossing plus three standard deviations lower right panel. PCA is rotate in 3D for better display. [file Image_1.TIF]
